# Supplementary material for: DNMT3A clonal hematopoiesis-driver mutations induce cardiac fibrosis by paracrine activation of fibroblasts
Source: Nat Commun. 2024 Jan 19;15:606. doi: 10.1038/s41467-023-43003-w (PMC10799021; doi:10.1038/s41467-023-43003-w)
Supplement: Supplementary file 1 — Supplementary Information File [file 41467_2023_43003_MOESM1_ESM.pdf]

Supplementary Figure 1

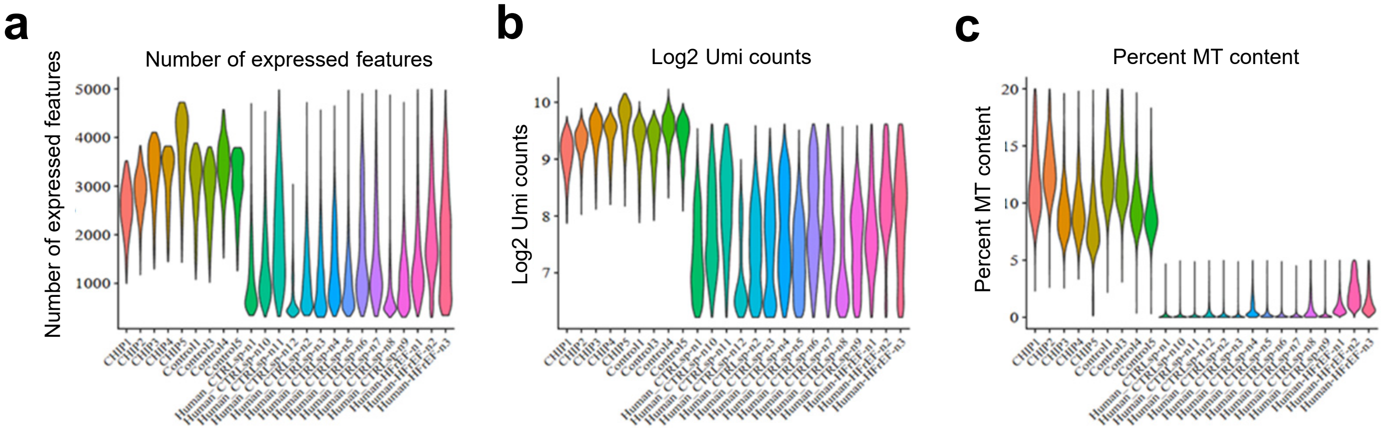

Supplementary figure 1. Quality control of integrated snRNA- and scRNA-sequencing data.

a. Violin plots depicting the number of expressed features **b**. UMI counts and percentage of c. mitochondrial content in individual samples.

Supplementary Figure 2

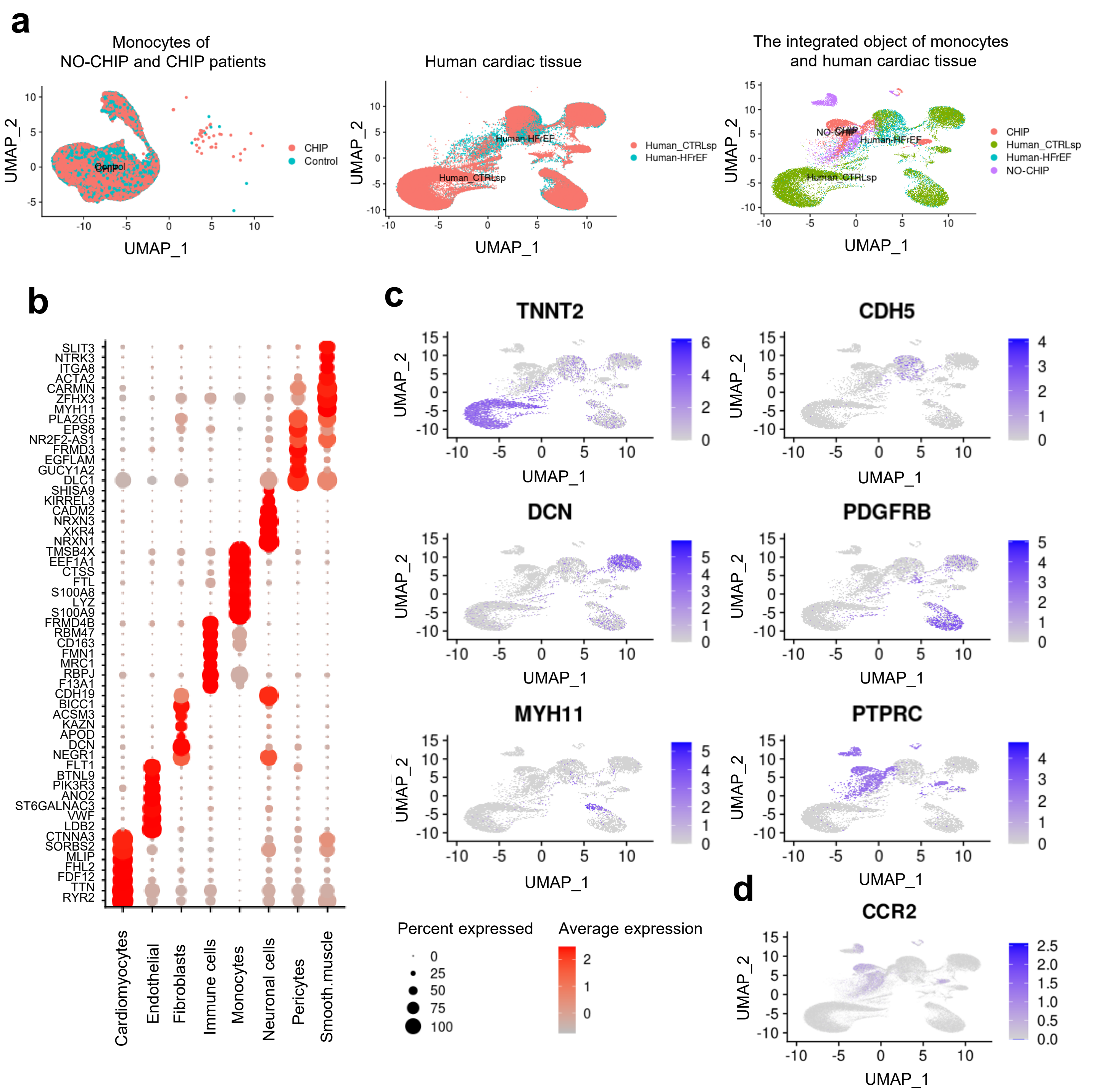

Supplementary Figure 2. scRNA-seq and scnRNA-seq integration quality control and cell annotation

a. Representative uniform manifold approximation and projection (UMAP) plots before and after integration of scRNA-seq dataset from PBMC obtained from HF patients with DNMT3A CHIP and without CHIP. Left, monocytes dataset from PBMCs of No-CHIP and DNMT3A CHIP patients before integration. Middle, human healthy and HFrEF cardiac tissue dataset before integration. Right, the integrated object of the monocytes and human cardiac tissue. **b.** Dot plot depicting representative marker gene expression in each cluster. **c.** Feature plots depicting gene expression of established cell-type specific marker genes: *TNNT2* for cardiomyocytes, *CDH5* for endothelial cells, *DCN* for fibroblasts, *PDGFRB* for pericytes, *MYH11* for smooth muscle cells, *PTPRC* for leukocytes. **D.** UMAP plot showing *CCR2* expression.

Supplementary Figure 3

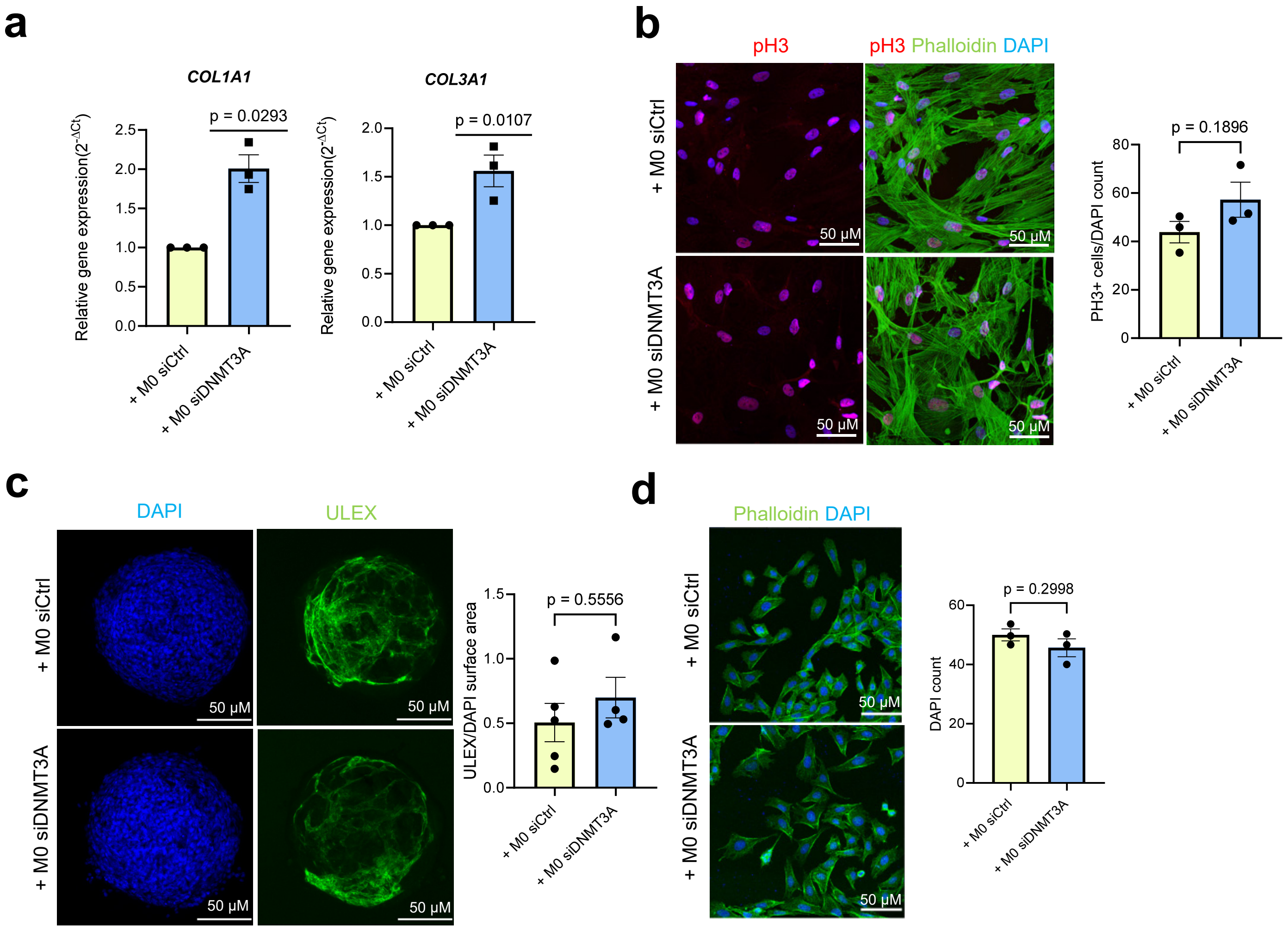

Supplementary Figure 3. Effects of supernatants of DNMT3A silenced monocytes

a. qPCR analysis of *COL1A1* and *COL3A1* gene expression in HCF treated with DNMT3A-silenced THP-1 monocytes supernatants (n=3 biologically independent experiments). Source data are provided as a Source Data file. **b.** Immunofluorescence analysis of pH3 in HCF treated with DNMT3A-silenced THP-1 monocytes supernatants (n=3 biologically independent experiments). Source data are provided as a Source Data file. **c.** Immunofluorescence analysis of ULEX in cardiospheres treated with DNMT3A-silenced THP-1 monocytes supernatants (n=5 for control and n=4 for siDNMT3A as biologically independent experiments). Source data are provided as a Source Data file. **d.** Analysis of cardiomyocyte number of cells treated with DNMT3A-silenced THP-1 monocytes supernatants (n=3 biologically independent experiments). Source data are provided as a Source Data file. Data are shown as mean ± SEM (**a-d**). Normal distribution was assessed using the Shapiro–Wilk test (**a-d**). Statistical analysis for comparison was performed using two-sided one sample t-tests (**a**) and using unpaired, two-sided Student's t-tests (**b, d**). t=5.710, 2 degrees of freedom; t=9.573, 2 degrees of freedom (**a**); t=1.578, 4 degrees of freedom (**b**); t=1.190, 4 degrees of freedom (**d**); Statistical analysis was performed using two-tailed Mann–Whitney test (**c**).

Supplementary Figure 4

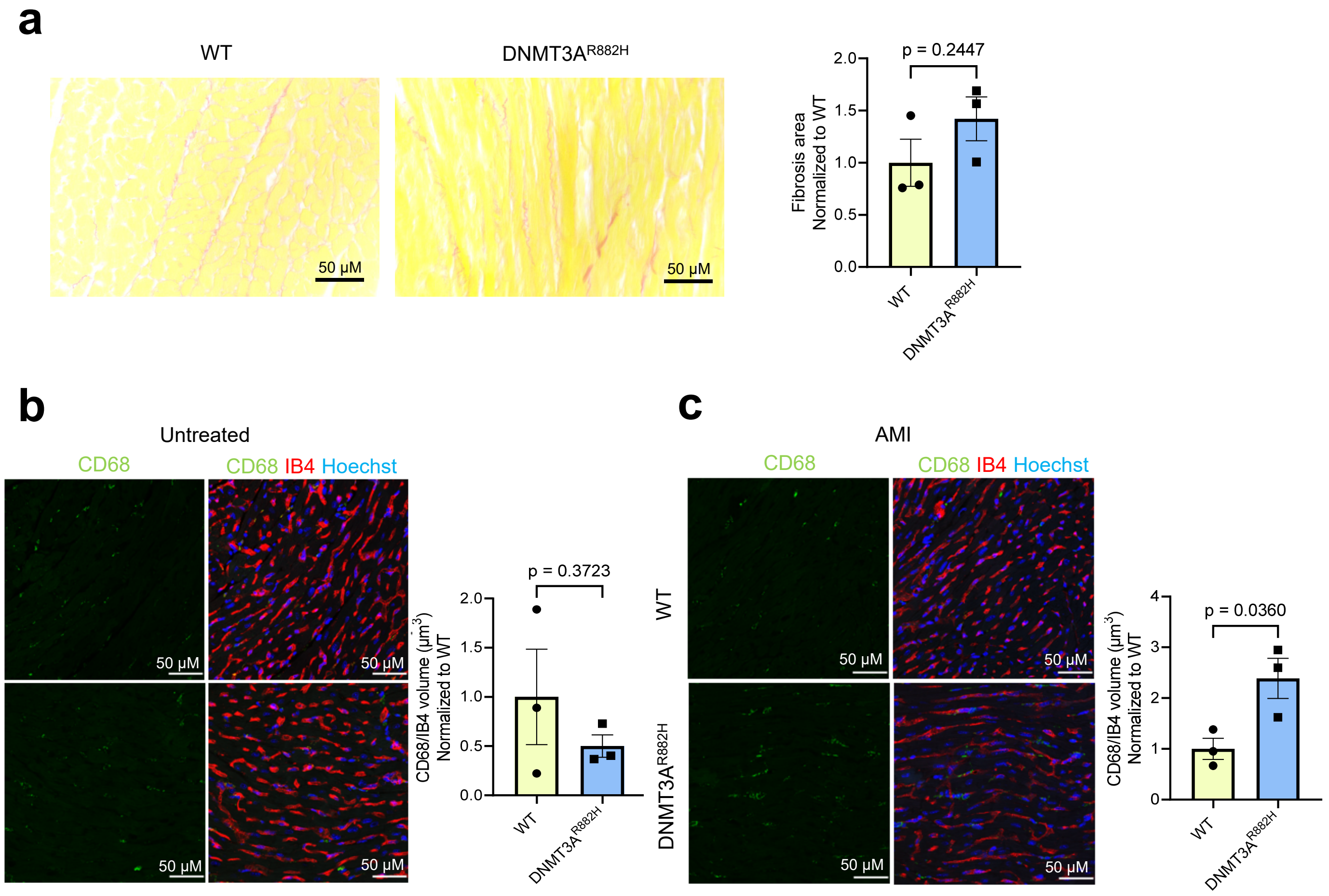

Supplementary Figure 4. Characterization of cardiac tissue from uninjured and infarcted WT and DNMT3A^{R882H} mice. a. Analysis of diffuse fibrosis using Picrosirius red staining in cross section of the cardiac septum in WT and DNMT3A^{R882H} mice (n=3 biologically independent experiments) (without infarction). Source data are provided as a Source Data file. **b.** Immunofluorescence analysis of CD68+ immune cell infiltration in the septum in untreated WT and DNMT3A^{R882H} mice (n=3 biologically independent experiments). Source data are provided as a Source Data file. **c** Immunofluorescence analysis of CD68+ immune cell infiltration in the remote zone of WT and DNMT3A^{R882H} mice after AMI (n=3 biologically independent experiments). Source data are provided as a Source Data file. Data are shown as mean ± SEM (**a-c**). Normal distribution was assessed using the Shapiro–Wilk test (**a-c**). Statistical analysis for comparison was performed using unpaired, two-sided Student's t-tests (**a-c**). t=1.363, 4 degrees of freedom (**a**); t=1.004, 4 degrees of freedom (**b**); t=1.363, 4 degrees of freedom (**c**).

Supplementary Figure 5

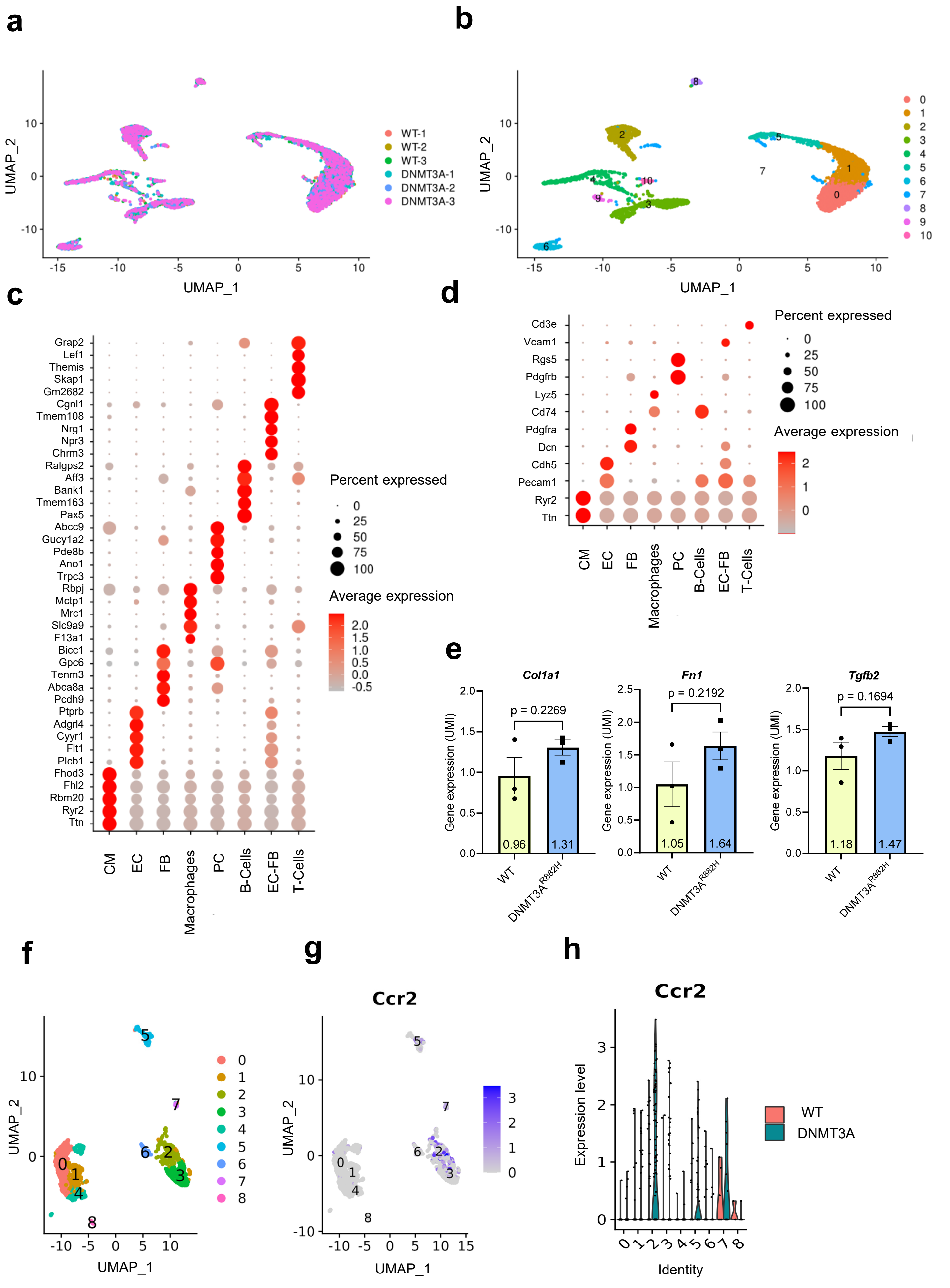

Supplementary Figure 5. Results of single nuclei RNA sequencing of WT and DNMT3A^{R882H} mice

a. Mice were exposed to infarction and hearts were collected 74-76 days post infarction for single nuclei RNA sequencing (n=3 biologically independent samples). Uniform manifold approximation and projection (UMAP) plots representing individual WT and DNMT3A^{R883H} hearts. Individual samples are color coded. **b**. UMAP plot representing the different cell type clusters identified after integration **c.** Dot plot of representative marker gene expression in each cell type cluster **d.** Dot plot of representative marker gene expression of established cell-type specific marker genes in each cell type cluster. CM, cardiomyocytes; EC, endothelial cells; FB, fibroblasts; PC, pericytes **e.** Mean expression of *Col1a1*, *Fn1*, and *Tgfb2* in WT and DNMT3A^{R882H} in fibroblasts of the hearts after AMI (n=3 biologically independent samples). Data are shown as mean ± SEM. Normal distribution was assessed using the Shapiro–Wilk test. Statistical analysis was performed using unpaired, two-sided Student's t-tests. t=1.426, 4 degrees of freedom; t=1.456, 4 degrees of freedom; t=1.674, 4 degrees of freedom. Source data are provided as a Source Data file. **f.** UMAP plots representing macrophages (subclusters 2, 3, 5, 6, 7) and fibroblasts (subclusters 0, 1, 4, 8) in WT and DNMT3A^{R882H} hearts (n=3 biologically independent samples). The different subclusters are color coded. **g.** UMAP plot showing Ccr2 expression in the different subclusters of macrophages (subclusters 2, 3, 5, 6, 7) and fibroblasts (subclusters 0, 1, 4, 8). **h.** Violin plots showing Ccr2 expression in the different subclusters of macrophages (subclusters 2, 3, 5, 6, 7) and fibroblasts (subclusters 0, 1, 4, 8). The genotype is color coded.

Supplementary Figure 6

Patient ID	Mutation	Mutation	VAF(%)
1	c.2644C>T	p.Arg882Cys	14,1
2	c.886G>A	p.Val296Met	2,04
3	c.2330C>T	p.Pro777Leu	2,12
4	c.1447G>A	p.Val483Met	2,45
5	c.2158C>T	p.Arg720Cys	1,14
6	c.748_754del	p.Pro250Thrfs*64	3,21
	c.2560G>T	p.Glu854*	1,79

Supplementary Figure 6. DNMT3A mutations in the 6 HF patients in the cMRI cohort; VAF - variant allele frequency.

Supplementary Figure 7

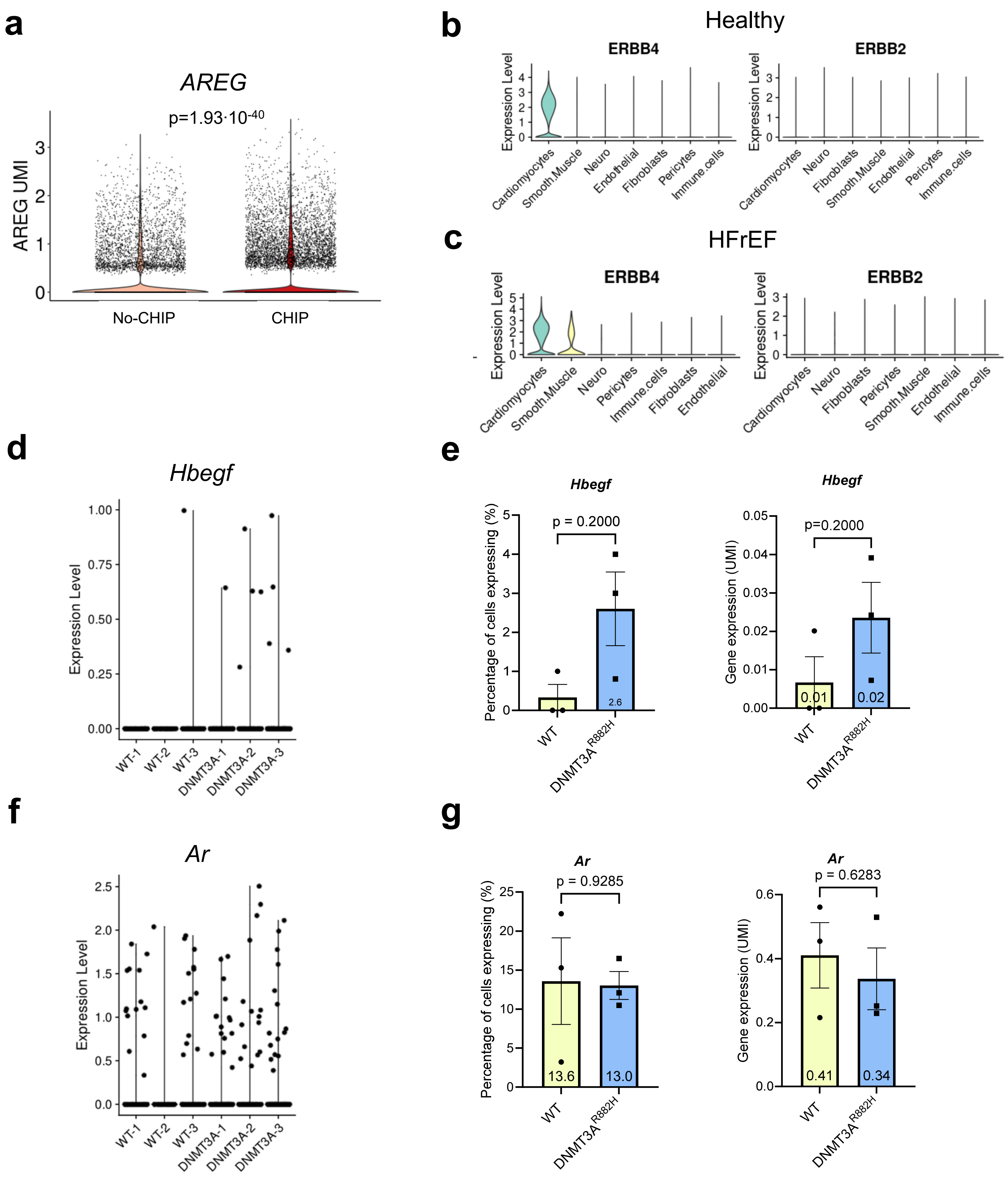

Supplementary Figure 7. DNMT3A CHIP monocytes/macrophages interact with fibroblasts through EGFR pathway.

a. Violin plot showing *AREG* gene expression in monocytes from No-CHIP and DNMT3A CHIP patients **b, c.** Violin plots depicting *ERBB4* and *ERBB2* gene expression in all the different cardiac cell types **b.** Healthy **c.** HFrEF **d.** Violin plot representing *Hbegf* gene expression in the immune cell cluster of hearts derived from WT and DNMT3A^{R882H} mice after AMI. **e.** Percentage of cells expressing *Hbegf* and *Hbegf* mean expression in the immune cell cluster of hearts from WT and DNMT3A^{R882H} mice after AMI (n=3 biologically independent samples). Source data are provided as a Source Data file. **f.** Violin plot represeting *Ar* (Areg) gene expression in the immune cell cluster from WT and DNMT3A^{R882H} mice after AMI **g.** Percentage of cells expressing *Ar* and *Ar* mean expression in immune cell clusters in hearts of WT and DNMT3A^{R882H} mice after AMI (n=3 biologically independent samples). Source data are provided as a Source Data file. Data are shown as mean ± SEM (**e,g**). Normal distribution was assessed using the Shapiro–Wilk test (**e,g**). Statistical analysis was performed using two-tailed Mann–Whitney test for **e** and unpaired, two-sided Student’s t-tests for **g**. t=0.09558, 4 degrees of freedom; t=0.5235, 4 degrees of freedom (**e**).

Supplementary Figure 8

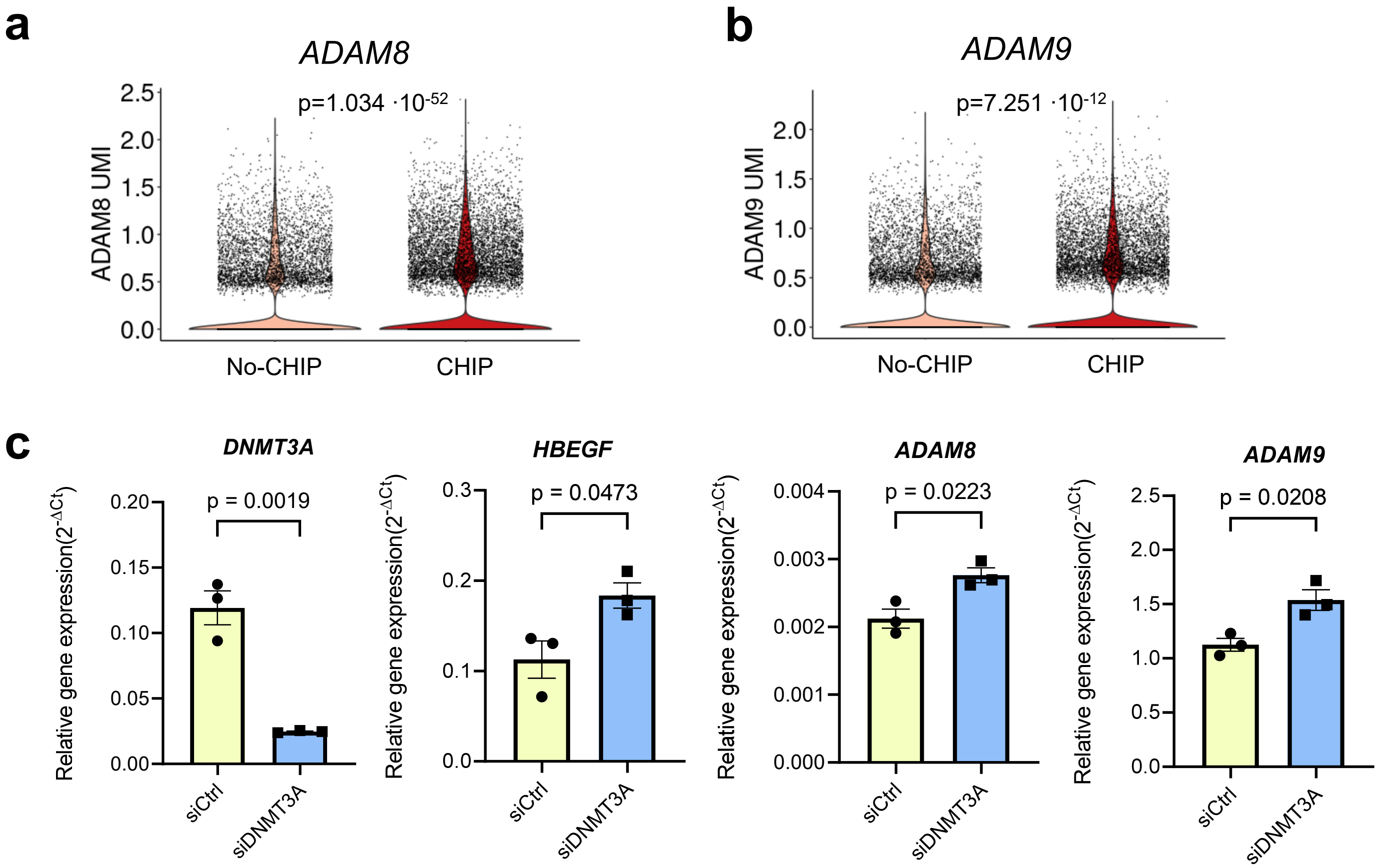

Supplementary Figure 8. Gene expression of *HBEGF* and *HBEGF*-activating genes

a, b. Violin plots showing *ADAM8* and *ADAM9* gene in monocytes from No-CHIP and DNMT3A CHIP HF patients. **c.** Transcript expression analysis by qPCR of *DNMT3A, HBEGF*, *ADAM8* and *ADAM9* in DNMT3A-silenced THP-1 monocytes (n=3 biologically independent samples). Source data are provided as a Source Data file. Data are shown as mean ± SEM (**c**). Normal distribution was assessed using the Shapiro–Wilk test (**c**). Statistical analysis for comparison of two groups was performed using unpaired, two-sided Student's t-tests (**c**). t=7.301, 4 degrees of freedom; t=2.831, 4 degrees of freedom; t=3.625, 4 degrees of freedom; t=3.704, 4 degrees of freedom (**c**).

Supplementary Figure 9

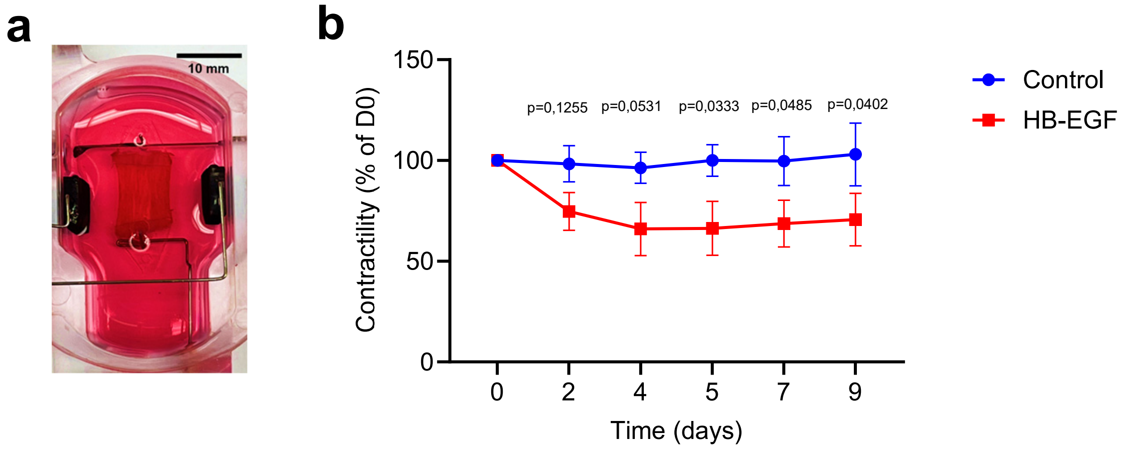

Supplementary Figure 9. Contractility of human heart slices treated with HB-EGF

a. Schematic of the human heart slice **b.** Contractility of human heart slices after treatment with HB-EGF (100 ng/ml, every 2. day) over 9 days (n=3 biologically independent samples per group). Source data are provided as a Source Data file. Data are shown as mean ± SEM (**b**). Normal distribution was assessed using the Shapiro–Wilk test (**b**). Statistical comparison was performed using two-way ANOVA with Fisher's LSD multiple comparisons posttest (**b**).

Supplementary Figure 10

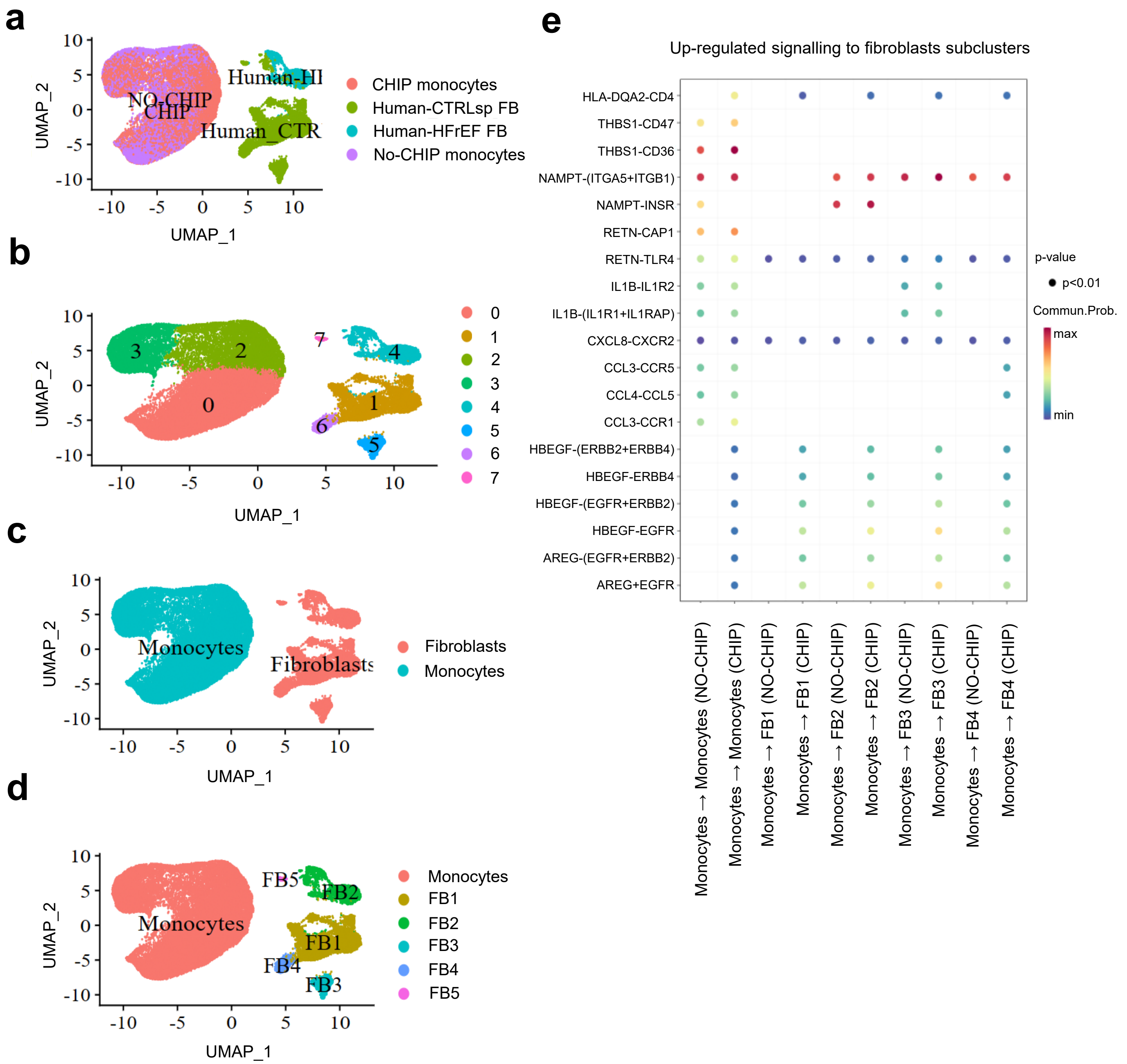

Supplementary Figure 10. Heterogeneity of human cardiac fibroblasts in the integrated object

a-d. Uniform manifold approximation and projection (UMAP) plots showing integration of DNMT3A CHIP and No-CHIP monocytes and subclusters of cardiac fibroblasts from healthy and HFrEF hearts **a.** Represents the different origin of the cells **b.** Different cellular clusters **c.** Cell annotation **d.** Subcluster annotation **e.** Bubble plots representing CHIP-upregulated ligand-receptor pairs in monocytes-to-fibroblasts subclusters. Color encodes communication probability, min. logFC for the interaction depicted is 0.1 and detection in minimum 10% of the cells.

Supplementary Table 1. Baseline characteristics of the HFrEF cohort

Baseline characteristics

	Sex	HFrEF	HFrEF	HFrEF
1	Age (years)	67	52	70
2	Sex	male	male	male
3	Ejection fraction (%)	12	29	40
4	NYHA class	3	3	3
5	Hypertension	none	none	none
6	Aortic valve stenosis	yes	none	none
7	History of moking	none	none	none
8	Dyslipidemia	none	none	none
10	Chronic heart disease	yes	yes	yes
11	Myocardial infarction	none	yes	yes
12	ACE inhibitor	none	none	none
13	AT1 blocker	yes	none	none
14	Betablocker	yes	yes	yes
15	Aspirin	yes	yes	yes
16	Statin	yes	none	yes

HFrEF, heart failure with reduced ejection fraction (HFrEF); NYHA, New York Heart Association.

Supplementary Table 2. Baseline characteristics of the cMRI study cohort

Baseline characteristics

	Characteristic	Total cohort (n = 38)	No-CHIP (n = 32)	CHIP (n = 6)	P value (CHIP versus NO CHIP)
1	Age, mean (SD) (n = 38)	66.34 (9.49)	66.25 (9.99)	66.83 (6.85)	0.8923
2	Male, No. (%) (n = 38)	30 (78.95)	26 (81.25)	4 (66.67)	0.587
3	Weight (kg), mean (SD) (n = 38)	82.47 (16.17)	82.81 (16.15)	80.67 (17.70)	0.77
4	Size (cm), mean (SD) (n = 38)	174.1 (10.96)	173.5 (10.93)	177.5 (11.52)	0.416
5	Hypertension, No. (%) (n = 34)	32 (94.12)	27 (96.43)	5 (83.33)	0.3262
6	Diabetes mellitus, No. (%) (n = 14)	6 (42.86)	4 (40.00)	2 (50.00)	>0.9999
7	Smoking, No. (%) (n = 36)	24 (66.67)	19 (63.33)	5 (83.33)	0.6399
8	Valve disease, No. (%) (n = 32)	6 (18.75)	5 (18.52)	1 (20.00)	>0.9999
9	Family history of coronary artery disease, No. (%) (n = 14)	7 (36.84)	6 (42.86)	1 (20.00)	0.6027
10	Coronary artery disease, No. (%) (n = 35)	16 (45.71)	14 (48.28)	2 (33.33)	0.4227
11	Myocardial infarction, No. (%) (n = 36)	7(19.44)	5 (16.67)	2 (33.33)	0.5732
12	Myocarditis, No. (%) (n = 35)	1 (2.86)	0 (0)	1 (16.67)	0.1714
13	NYHA class, mean (SD) (n=33)	1.818 (0.7687)	1.926 (0.7808)	1.333 (0.5164)	0.1056
14	NT-proBNP serum levels (pg/ml), mean (SD) (n = 31)	8869 (18763)	7540 (16540)	14406 (27450)	0.7206
15	High sensitivity C-reactive protein levels (mg/dl), mean (SD) (n = 31)	38.45 (64.43)	43.77 (69.15)	10.8 (10.5)	0.1466
16	High sensitive troponin T levels (pg/ml), mean (SD) (n = 32)	137.1 (294.3)	123 (289)	197.8 (338)	0.1768
17	Hemoglobin (g/dl), mean (SD) (n = 33)	128.4 (41.65)	133.4 (37.91)	105.5 (53.50)	0.1109
18	Hematocrit, mean (SD) (n = 33)	374.8 (120.2)	392.6 (109.3)	295.2 (144.8)	**0.0497**
19	Thrombocytes (/µl), mean (SD) (n = 32)	245.2 (63.83)	235.8 (61.47)	285.8 (62.65)	0.1055
20	Leukocytes (/µl), mean (SD) (n = 33)	618.3 (267.9)	677 (211.9)	354 (351.5)	**0.0055**
21	ACE inhibitor /AT1 blocker, No. (%) (n = 32)	18 (56.25)	13 (50.00)	5 (83.33)	0.1959
22	Aldosterone blocker, No. (%) (n = 32)	18 (56.25)	14 (53.85)	4 (66.67)	0.6722
23	Betablocker, No. (%) (n = 32)	27 (84.38)	21 (80.77)	6 (100)	0.5546
24	Statin, No. (%) (n = 32)	21 (65.63)	17 (65.38)	4 (66.67)	>0.9999
25	Diuretics, No. (%) (n = 32)	13 (40.63)	9 (34.62)	4 (66.67)	0.1937

Data are shown as mean ± SD (1, 3, 4, 13-20) or as counts (percentage of the group, %) (2, 5-12, 21-25). Normal distribution was assessed using the Shapiro–Wilk test (1, 3, 4, 13-20). Statistical analysis for the comparison of

two groups was performed using a two-tailed Mann–Whitney test for data not following a Gaussian distribution and an unpaired, two-sided Student's t-tests (1, 3, 4, 13-20). Fischer exact tests were used for proportions (2, 5-12, 21-25).

NYHA, New York Heart Association; NT-proBNP, N-terminal-pro-Brain Natriuretic Peptide.

Supplementary Table 2. Baseline characteristics of the cMRI study cohort

cMRI findings

	Characteristic	Total Cohort (n = 38)	No-CHIP (n = 32)	CHIP (n = 6)	P value (CHIP versus NO CHIP)
1	LVEF (%), mean (SD) (n = 35)	34.06 (9.11)	34.83 (9.22)	30.33 (8.287)	0.5561
2	LV-EDVi (mL/m2), mean (SD) (n = 33)	113.6 (40.14)	111.1 (40.3)	124.5 (41.15)	0.4699
3	LV mass index (g/m2), mean (SD) (n = 32)	73.03 (31.53)	75.23 (33.76)	63.5 (18.2)	0.3864
4	RVEF (%), mean (SD) (n = 34)	43.26 (11.14)	44.18 (11.49)	39 (8.922)	0.3085
5	Native T1 (ms), mean (SD) (n = 33)	1146 (96.64)	1131 (97.46)	1214 (61.15)	**0.0497**
6	Native T2 (ms), mean (SD) (n = 33)	39.42 (4.10)	39.41 (4.00)	39.5 (4.93)	0.7754
7	Pericardial effusion, No. (%) (n = 35)	11 (31.42)	8 (26.67)	3 (60.00)	0.297
8	LGE, No (%) (n = 30)	21 (70.00)	17 (70.83)	4 (66.67)	>0.9999

Data are shown as mean ± SD (1-3, 5-6) or as counts (percentage of the group, %) (7-8). Normal distribution was assessed using the Shapiro–Wilk test (1-3, 5-6). Statistical analysis for the comparison of two groups was performed using a two-tailed Mann–Whitney test for data not following a Gaussian distribution and an unpaired, two-sided Student’s t-tests (1-3, 5-6). Fischer exact tests were used for proportions (7-8).

LVEF, left ventricular ejection fraction; LV- EDVi, left ventricular end-diastolic volume, indexed to body surface area; LV - left ventricular; RVEF, reduced right ventricular ejection fraction; LGE, late gadolinium enhancement.

Supplementary Table 3. Baseline characteristics of the serum study cohort

	Characteristic	Total Cohort (n = 20)	No-CHIP (n = 10)	CHIP (n = 10)	P value (CHIP versus NO CHIP)
1	Age, mean (SD) (n = 20)	67.25 (11.15)	66.30 (11.95)	68.2 (10.84)	0.714
2	Male, No. (%) (n = 20)	18.00 (90.00)	9.00 (90.00)	9.00 (90.00)	>0.9999
3	Weight (kg), mean (SD) (n = 39)	83.60 (17.30)	88.73 (20.75)	77.90 (10.77)	0.1788
4	Size (cm), mean (SD) (n = 17)	171.60 (7.86)	172.20 (9.01)	171.00 (6.89)	0.7601
5	Hypertension, No. (%) (n = 18)	12.00 (66.67)	5.00 (55.56)	7.00 (77.78)	0.6199
6	Diabetes mellitus, No. (%) (n = 20)	10.00 (50.00)	5.00 (50.00)	5.00 (50.00)	>0.9999
7	Smoking, No. (%) (n = 19)	11.00 (57.89)	5.00 (50.00)	6.00 (66.67)	0.6499
8	Valve disease, No. (%) (n = 17)	2.00 (11.76)	2.00 (18.18)	0.00 (0.00)	0.4854
9	Coronary artery disease, No. (%) (n = 20)	19.00 (95.00)	9.00 (90.00)	10.00 (100.00)	>0.9999
10	Myocardial infarction, No. (%) (n = 20)	12.00 (60.00)	6.00 (60.00)	6.00 (60.00)	>0.9999
11	NYHA class, mean (SD) (n=13)	1.89 (0.92)	1.80 (1.09)	1,94 (0.86)	0.9068
12	LVEF (%), No. (%) (n = 20)	38.50 (9.61)	39.00 (9.37)	38.00 (10.33)	0.7799
13	NT-proBNP serum levels (pg/ml), mean (SD) (n = 20)	941.07 (811.80)	836.20 (821.10)	1047.00 (832.00)	0.4935
14	Hemoglobin (g/dl), mean (SD) (n = 33)	13.35 (1.96)	13.76 (1.20)	12.94 (2.51)	0.3635
15	Thrombocytes (/µl), mean (SD) (n = 20)	446.80 (801.20)	240.80 (62.42)	652.80 (1121.00)	0.255
16	Leukocytes (/µl), mean (SD) (n = 20)	8.57 (2.28)	8.06 (2.76)	9.08 (1.66)	0.3297
17	Renin-Angiotensin-Aldosterone blockade - ACE inhibitor /AT1 blocker, No. (%) (n = 20)	14.00 (70.00)	8.00 (80.00)	6.00 (60.00)	0.6285
18	Renin-Angiotensin-Aldosterone blockade - Aldosterone blocker, No. (%) (n = 20)	10.00 (50.00)	4.00 (40.00)	6.00 (60.00)	0.6563
19	Betablocker, No. (%) (n = 20)	19.00 (95.00)	9.00 (90.00)	10.00 (100.00)	>0.9999
20	Statin, No. (%) (n = 32)	20.00 (100.00)	10.00 (100.00)	10.00 (100.00)	>0.9999

Data are shown as mean ± SD (1,3-4; 11; 13-16) or as counts (percentage of the group, %) (2,5-10,17-20). Normal distribution was assessed using the Shapiro–Wilk test (1,3-4; 11; 13-16). Statistical analysis for the comparison of two groups was performed using a two-tailed Mann–Whitney test for data not following a Gaussian distribution and an unpaired, two-sided Student's t-tests (1,3-4; 11; 13-16). Fischer exact tests were used for proportions (2,5-10,17-20).

NYHA, New York Heart Association; LVEF, left ventricular ejection fraction; NT-proBNP, N-terminal-pro-Brain Natriuretic Peptide.
