# Supplementary material for: DNMT3A clonal hematopoiesis-driver mutations induce cardiac fibrosis by paracrine activation of fibroblasts
Source: Nat Commun. 2024 Jan 19;15:606. doi: 10.1038/s41467-023-43003-w (PMC10799021; doi:10.1038/s41467-023-43003-w)
Supplement: Supplementary file 3 — Reporting Summary [file 41467_2023_43003_MOESM3_ESM.pdf]

Corresponding author(s): Stefanie Dimmeler

Last updated by author(s): Oct 24, 2023

Reporting Summary

Nature Portfolio wishes to improve the reproducibility of the work that we publish. This form provides structure for consistency and transparency in reporting. For further information on Nature Portfolio policies, see our Editorial Policies and the Editorial Policy Checklist.

Statistics

For all statistical analyses, confirm that the following items are present in the figure legend, table legend, main text, or Methods section.

n/a	Confirmed	
☐	☒	The exact sample size (*n*) for each experimental group/condition, given as a discrete number and unit of measurement
☐	☒	A statement on whether measurements were taken from distinct samples or whether the same sample was measured repeatedly
☐	☒	The statistical test(s) used AND whether they are one- or two-sided<br>*Only common tests should be described solely by name; describe more complex techniques in the Methods section.*
☒	☐	A description of all covariates tested
☐	☒	A description of any assumptions or corrections, such as tests of normality and adjustment for multiple comparisons
☐	☒	A full description of the statistical parameters including central tendency (e.g. means) or other basic estimates (e.g. regression coefficient) AND variation (e.g. standard deviation) or associated estimates of uncertainty (e.g. confidence intervals)
☐	☒	For null hypothesis testing, the test statistic (e.g. *F*, *t*, *r*) with confidence intervals, effect sizes, degrees of freedom and *P* value noted<br>*Give P values as exact values whenever suitable.*
☒	☐	For Bayesian analysis, information on the choice of priors and Markov chain Monte Carlo settings
☒	☐	For hierarchical and complex designs, identification of the appropriate level for tests and full reporting of outcomes
☒	☐	Estimates of effect sizes (e.g. Cohen's *d*, Pearson's *r*), indicating how they were calculated

Our web collection on statistics for biologists contains articles on many of the points above.

Software and code

Policy information about availability of computer code

Data collection	Data collection software used are described in the methods section. Skyra and Prisma, Siemens Healthineers software (version VE11, Siemens), Volocity (x64) software (version 6.5.1, Quorum Technologies, Canada), Leica Application Suite X 2.0.1.14392, NIS Elements (version 5.21, Nikon Instruments Software), Vevo3100 (Fujifilm VisualSonics), ImageLab (version 5.0, Bio-Rad), QuantStudio Software (version 1.3, Thermo Fischer Scientific), Axiovision (version 4.5, Zeiss).
Data analysis	Packages and software used are described in the methods section. We used custom code found at https://github.com/djhn75/Nat-Com-DNMT3A-Analysis. Medis Suite MR (version 2.1, Medis Medical Imaging Systems), Volocity (x64) software (version 6.5.1, Quorum Technologies, Canada), Prism 9.2.0 (GraphPad Software Inc.), ImageLab (version 5.0, Bio-Rad), ImageJ Fiji (version 2.3.1), QuantStudio Software (version 1.3, Thermo Fischer Scientific), Axiovision (version 4.5, Zeiss), Seurat 4, Cell Ranger Single Cell Software Suite 7.0.0, STARsolo (v 2.7.3a), CellChat (v 1.5.0).

For manuscripts utilizing custom algorithms or software that are central to the research but not yet described in published literature, software must be made available to editors and reviewers. We strongly encourage code deposition in a community repository (e.g. GitHub). See the Nature Portfolio guidelines for submitting code & software for further information.

Data

Policy information about availability of data

All manuscripts must include a data availability statement. This statement should provide the following information, where applicable:

- Accession codes, unique identifiers, or web links for publicly available datasets
- A description of any restrictions on data availability
- For clinical datasets or third party data, please ensure that the statement adheres to our policy

The single-nuclei RNA-Seq data generated in this study have been deposited in the at ArrayExpress Data Depository (https://www.ebi.ac.uk/biostudies/arrayexpress) with series accession number E-MTAB-13384. Following datasets from ArrayExpress Data Depository were reused in this manuscript: accession number E-MTAB-13016 (monocytes scRNA-seq dataset) and E-MTAB-13264 (human HFrEF snRNA-seq dataset). The used snRNA-seq dataset of the septum from healthy heart samples was taken from Heart Cell Atlas (version 2, https://www.heartcellatlas.org/#DataSources). Clinical data from Myoflame 19 study (EudraCT 2022-00162-12, NCT05619653) was reused in the publication. CellChat analysis package and related tools are accessible under https://github.com/sqjin/CellChat. Enrichr Database used in the manuscript could be found under https://maayanlab.cloud/Enrichr/. Associated Source Data file is provided within the article. All other data included in the manuscript, source data (with statistical tests performed), and supplementary data are provided in the main article and/or at the referenced depositories or are available upon request per e-mail: dimmeler@em.uni-frankfurt.de.
Licensed third-party art resource BioRender (28D5A348-0001) was used to create figures for publication.

Research involving human participants, their data, or biological material

Policy information about studies with human participants or human data. See also policy information about sex, gender (identity/presentation), and sexual orientation and race, ethnicity and racism.

Reporting on sex and gender	Sex of the human patients is reported in supplementary tables 1-3.
Reporting on race, ethnicity, or other socially relevant groupings	Social groupings have not been taken into account in this manuscript.
Population characteristics	Population characteristics are reported in supplementary tables 1-3 and in the Methods section.
Recruitment	For cardiac MRI study cohort, all consenting human research participants attended a visit for collection of clinical data, cardiac biomarkers and imaging at University Hospital Frankfurt (in the frame of Myoflame-19 study, EudraCT 2022-00162-12, NCT05619653). For the heart failure with reduced ejection fraction (HFrEF) patients with and without CHIP serum analysis, patients were recruited on an all-comers base in the outpatient heart failure clinic. Consenting human research participants were giving peripheral blood samples at the Department of Cardiology, University Frankfurt (in the frame of the UCT-Project-Nr.: KardioBMB#2022-004). No self-selection or other biases, that might affect the results, were present. For human myocardial slices study, all available tissues from consenting dilatory cardiomyopathy patients undergoing cardiac surgery have been included irrespective of gender or sex. Sex and/or gender of participants was determined based on self-report of the study participants.
Ethics oversight	Human studies comply with all relevant ethical regulations and were approved by the respective institutional ethics committees (cardiac magnetic resonance (CMR) imaging and post-processing (Fig. 5a-g, Supplementary Table 2), peripheral blood samples analysis (Fig. 6j, Supplementary Table 3) - Ethics Committee of University Hospital of the Johann Wolfgang Goethe University and the Johann Wolfgang Goethe University, Frankfurt am Main, Germany; preparation and treatment of human myocardial slices (Supplementary Fig. 9a, b) - Ethics Committee of the Ruhr-University Bochum and Erich & Hanna Klessmann-Institute, Ruhr University Bochum, Heart & Diabetes Center NRW, Bad Oeynhausen, Germany; Ethics Committee of the Ludwig-Maximilians-University Munich and Walter-Brendel-Centre of Experimental Medicine, University Hospital of the Ludwig-Maximilians-University, Munich, Germany). All participants provided written informed consent. All procedures were performed in concordance with internal standards of the German government, followed institutional guidelines, the Declaration of Helsinki and the International Conference on Harmonization of Good Clinical Practice. No compensation was provided to participants.

Note that full information on the approval of the study protocol must also be provided in the manuscript.

Field-specific reporting

Please select the one below that is the best fit for your research. If you are not sure, read the appropriate sections before making your selection.

☒ Life sciences ☐ Behavioural & social sciences ☐ Ecological, evolutionary & environmental sciences

For a reference copy of the document with all sections, see nature.com/documents/nr-reporting-summary-flat.pdf

Life sciences study design

All studies must disclose on these points even when the disclosure is negative.

Sample size	Required sample sizes for in vivo experiments were estimated using power-calculation (p=0.8). For in vitro experiments sample number was

Sample size	defined according to previous experience by us or others, which showed successful silencing of DNMT3A in monocytic cell line and significant increase in cardiac fibroblasts activation upon stimulation, and reproducibility of the results across several independent experiments.<br>e.g.<br>Abplanalp et al. Circulation Research 2021;216-228. doi:10.1161/CIRCRESAHA.120.317104 (n=3)<br>Nicin, Schroeter, Glaser et al. Nat Cardiovasc Res vol.1 2022, 174–185. doi: 10.1038/s44161-022-00019-7(n=3)<br>Cattaneo et al. Nat Com vol. 13 2022,1 7444 doi:10.1038/s41467-022-35070-2(n=3)
Data exclusions	No data was excluded.
Replication	All biological replicates performed were included within the data shown in the manuscript and are accordingly described in the legends. Individual values are shown in each figure.<br>For all in vivo experiments, the number of replicates is given in the respective figure legends. In vitro experiments were generally performed in at least 2 independent experiments to ensure reproducibility of the data. All samples were generated independently to ensure biological, and not technical, replications. For ex vivo experiments, measurements were performed in biologically independent experiments deriving from different donors.<br>We confirm that all attempts in replication were successful.
Randomization	Wild type animals have been randomly assigned to treatment cohorts. Transgenic mice were compared to their litter mates controls. Cells have been randomly allocated to respective groups. Ex vivo heart slices treatment was randomly assigned to respective groups.
Blinding	All single-cell and single-nuclei RNA-sequencing analysis was performed blindly, as unbiased published algorithms for f.ex. clustering and ligand-receptor analysis were used. Researchers were blind to patient mutation status, mouse genotypes and treatments for quantification in patient and in vivo studies. In vitro experiments were partially non-blinded due to poor feasibility and limited working force.

Reporting for specific materials, systems and methods

We require information from authors about some types of materials, experimental systems and methods used in many studies. Here, indicate whether each material, system or method listed is relevant to your study. If you are not sure if a list item applies to your research, read the appropriate section before selecting a response.

Materials & experimental systems

n/a	Involved in the study
☐	☒ Antibodies
☐	☒ Eukaryotic cell lines
☒	☐ Palaeontology and archaeology
☐	☒ Animals and other organisms
☒	☐ Clinical data
☒	☐ Dual use research of concern
☒	☐ Plants

Methods

n/a	Involved in the study
☒	☐ ChIP-seq
☒	☐ Flow cytometry
☒	☐ MRI-based neuroimaging

Antibodies

Antibodies used	Immunohistochemistry<br>Primary antibodies: biotinylated Isolectin-B4 (B-1205.5; Vector Laboratories) (1:25), rabbit anti-CD68 (97778S, Cell signaling) (1:100).<br>Secondary antibodies: Donkey anti rabbit Alexa Fluor 488 (A21206, Invitrogen) (1:200), Streptavidin Alexa Fluor 647 (S32357; Invitrogen) (1:200). Nuclei were counterstained using Hoechst (33342; Sigma-Aldrich) (1:1000).<br><br>Western blot<br>Primary antibodies: (1:1000 rabbit anti-DNMT3A (E9P2F), #49768, Cell Signaling; 1:1000 rabbit anti-Akt (pan) (C67E7), #4691, Cell Signaling; 1:1000 rabbit anti-phospho-Akt (Ser473) (193H12), #4058, Cell Signaling; 1:1000 rabbit anti-HBEGF (E5L5T), #27450, Cell Signaling; 1:1000 rabbit anti-α-tubulin Antibody, #2144, Cell Signaling). Secondary antibodies (1:1000 donkey anti-rabbit IgG, HRP-linked Antibody, #7074S, Cell Signaling).<br><br>Cardiac tissue mimetics immunofluorescence staining<br>Primary antibodies and dyes (mouse anti-α-smooth muscle actin (1:40, #C6168, Sigma-Aldrich), goat anti-PDGFRα (1:20, #AF1062, R&D Systems), biotinylated Ulex Europaeus Agglutinin I (1:100, #B-1065-2, Vector Laboratories), rabbit anti-COL1A1 (1:20, 72026S, Cell Signaling).<br>Secondary antibodies and dyes (streptavidin 488 (#S11223, Thermo Fisher Scientific), donkey anti-goat-647 (1:200, #A-21447, Thermo Fisher Scientific), anti-rabbit-555 (1:200, #A-21428, Thermo Fisher Scientific), anti-rabbit-647 (1:200, #A-31573, Thermo Fisher Scientific), DAPI (1:1000, #D9542, Merck)).<br><br>Immunofluorescence staining for cell culture<br>Primary antibodies and dyes (phalloidin (1:100, #O7466, Thermo Fisher Scientific), mouse anti-α -smooth muscle actin (1:200, #C6168, Sigma-Aldrich), rabbit anti-phospho-histone H3 (Ser10; 1:200, #06-570, Sigma-Aldrich)).<br>Secondary antibodies and dyes (donkey anti-rabbit-647 (1:200, #A-31573, Thermo Fisher Scientific), DAPI (1:1000, #D9542, Merck)).
Validation	Primary antibodies were validated by the manufacturer and confirmed by specific labeling of target molecules or cell types.

Validation

biotinylated Isolectin-B4 https://vectorlabs.com/products/glycobiology/biotinylated-gsl-i-isolectin-b4
rabbit anti-CD68 https://www.cellsignal.com/products/primary-antibodies/cd68-e3o7v-rabbit-mab/97778
rabbit anti-DNMT3A (E9P2F) https://www.cellsignal.com/products/primary-antibodies/dnmt3a-e9p2f-rabbit-mab/49768
rabbit anti-Akt (pan) (C67E7) https://www.cellsignal.com/products/primary-antibodies/akt-pan-c67e7-rabbit-mab/4691
abbit anti-phospho-Akt (Ser473) (193H12) https://www.cellsignal.com/products/primary-antibodies/phospho-akt-ser473-193h12-rabbit-mab/4058
rabbit anti-HBEGF (E5L5T) https://www.cellsignal.com/products/primary-antibodies/hbegf-e5l5t-rabbit-mab/27450
rabbit anti-α-tubulin https://www.cellsignal.com/products/primary-antibodies/a-tubulin-antibody/2144?gclid=Cj0KCQjw756lBhDMARIsAEI0Agn0pvvlH5RbDL-EvH_DnUPxlu1VYnSmPYgY_jLDFZK6VE1oLvdXIKgaAsvwEALw_wcB&gclsrc=aw.ds
goat anti-PDGFRα https://www.rndsystems.com/cn/products/mouse-pdgf-ralpha-antibody_af1062?gclid=Cj0KCQjw756lBhDMARIsAEI0AgnrTSWNQgHvz5e2Iv7sZLJAN6DdR-sCedRn_QP9Hgt_5rgJ5crW054aAqELEALw_wcB&gclsrc=aw.ds
biotinylated Ulex Europaeus Agglutinin I https://vectorlabs.com/products/biotinylated-ulex-europaeus-agglutinin.html
mouse anti-α -smooth muscle actin https://www.sigmaaldrich.com/DE/en/product/sigma/c6198?gclid=Cj0KCQjw756lBhDMARIsAEI0Aglue4ygqmit_e9QJpw9ExNcYimCG28OhvBZmcGAUc0FslVKyb4_aX8aArapEALw_wcB&gclsrc=aw.ds
rabbit anti-phospho-histone H3 https://www.sigmaaldrich.com/DE/en/product/mm/06570?gclid=Cj0KCQjw756lBhDMARIsAEI0Agn-RYMkaJ4xhjnn6VqbFqmXspludwaaODif9hClTItHj_DRd3FX-x4aAoQNEALw_wcB&gclsrc=aw.ds
rabbit anti-COL1A1 (E8F4L) https://www.cellsignal.com/products/primary-antibodies/col1a1-e8f4l-xp-rabbit-mab/72026

Secondary antibodies have been tested in our experimental conditions to rule out unspecific reactivity.

Eukaryotic cell lines

Policy information about cell lines and Sex and Gender in Research

Cell line source(s)	THP-1 (#ACC16, DSMZ), Primary human cardiac fibroblasts (HCF) (#C-12375, Promocell), immortalized human cardiac fibroblasts (iHCF) (#P10453-IM, Innoprot), Human umbilical vein endothelial cells (HUVECs) (#C-12203 / C-12253, PromoCell), human cardiomyocyte ventricular primary cells (HCM-VT) (#36044-15VT)
Authentication	In this study we only used low passages of commercially available cell lines. We did not include extra authentication steps.
Mycoplasma contamination	All cells in this is study have been routinely tested for mycoplasma contamination and all experiments have been done on mycoplasma free cells.
Commonly misidentified lines (See ICLAC register)	ICLAC register (version 12.0) did not list used cell lines as commonly misidentified lines.

Animals and other research organisms

Policy information about studies involving animals; ARRIVE guidelines recommended for reporting animal research, and Sex and Gender in Research

Laboratory animals

DNMT3AR882H knock-in (KI) mice were generated by the Mouse Biology Program (MBP) of the University of California (Davis, CA) as previously described34. The DNMT3Afl-R882H-fl mice were crossed to B6.Cg-Tg(Mx1-cre)1Cgn/J mice (referred to as Mx1-Cre) or to tamoxifen-inducible transgenic B6.129-Gt(ROSA)26Sortm1(cre/ERT2)Tyj/J mice (referred to as R26-CreERT2 Cre recombinase - estrogen receptor T2). C57BL/6N (CD45.2+ or Ly5.2+) and B6.SJL-PtprcaPepcb/BoyJ (CD45.1+ or Ly5.1+) mice were obtained from Charles River Laboratory. Primary bone marrow cells of age-matched 16- to 28-week old female and male Mx1-Cre+/DNMT3A+/R882H and Mx1-Cre-/DNMT3A+/+ donor mice (Ly5.2+) were collected 14 days after the last pI:pC injection (Amersham; 400µg/mouse intraperitoneally for 3 nonconsecutive days) to ensure that signaling activation and cytotoxic effects mediated by pI:pC were minimized. To validate excision efficiency, genomic DNA from blood or harvested cells was subjected to PCR.
12- to 16-week old female recipient mice were lethally (7.5 Gy total body irradiation) irradiated using X-Ray Irradiation System MultuRad 160 (Faxitron Bioptics LLC, USA). Recipient mice were maintained on Cyprofloxacin drinking water (40/mg/kg, Fresenius Kabi Deutschland GmbH, Bad Homburg) for one week before and two weeks after transplantation and allowed to engraft for 6 weeks, before being used for analysis. For transplantation, donor bone marrow cells from primary DNMT3A+/R882H (Ly5.2+) or wild-type +/+ (Ly5.2+) mice were transplanted into lethally irradiated Ly5.1 (B6.SJL-Ptprca Pepcb/BoyJ; Charles River Laboratory) recipients with a total of 2.5 x 105 cells per recipient. Peripheral blood engraftment was assessed by flow cytometry 6 weeks post transplantation.
Six weeks after reconstitution of the bone marrow, myocardial infarction was induced by permanent ligation of the left anterior descending artery in transplanted recipients. In brief, anesthesia was induced with isoflurane (4%/800 ml O2/min) and maintained by endotracheal ventilation (2–3%/800 ml O2/min). Thoracotomy was performed in the fourth left intercostal space. The left ventricle was exposed, and the left coronary artery was permanently occluded. Chest and skin were closed, and anesthesia was terminated. Animals were extubated when breathing was restored. Initial myocardial injury was evaluated by measuring cardiac Troponin T levels in plasma 24 h after induction of myocardial infarction. To obtain hearts after four weeks, mice were sacrificed via cervical dislocation during isoflurane anesthesia and perfused with cold Hank's buffered saline solution (HBSS, 14175-053, Invitrogen).
For mouse single-cell nuclei (sn) and single-cell (sc) RNA sequencing we used a transplantation model with tamoxifen-inducible Rosa26CreERT2:DNMT3A-R882H (Cre recombinase - estrogen receptor T2; known as R26-CreERT2 strain) intercross mice as donors.
For mouse single-cell nuclei (sn) and single-cell (sc) RNA sequencing we used a transplantation model with tamoxifen-inducible Rosa26CreERT2:DNMT3A-R882H (Cre recombinase - estrogen receptor T2; known as R26-CreERT2 strain) intercross mice as donors.
These mice do not require pI:pC treatment and are independent of intrinsic inflammatory IFN-response to induce DNMT3A-R882H expression within the hematopoietic compartment.

We generated complete "chimeras" mice by transplanting of non-induced Rosa26CreERT2:DNMT3A-R882H (Ly5.1+) hematopoietic cells (either (+/+) or (+/m), 16-28-week old, female and male) into 16-week old female Ly5.1/2 recipients. 6 weeks after BM cells transplantation, recipients were subjected to myocardial infarction as described above. Dissolved tamoxifen (Sigma-Aldrich) in corn oil was administered via intraperitoneal injection (1 mg/mouse/day) once every 24 hours for a total of 10 consecutive days. The injections started 28 days after the induction of the myocardial infarction. The mice were used at day 74-76 post left anterior descending artery ligation through sacrifice via cervical dislocation during isoflurane anesthesia.
All mice were housed under standard conditions in individually ventilated cages with controlled dark–light cycle, temperature and humidity in cages at the animal facility of Heidelberg University Hospital according to national and institutional guidelines for animal care.
For the isolation of neonatal rat cardiomyocytes and fibroblasts and cardiospheres generation, mated female Sprague Dawley rats (>12 weeks old) were obtained from Janvier Labs and housed under standard conditions with controlled dark–light cycle, temperature and humidity in cages at the animal facility of University Hospital of the Johann Wolfgang Goethe University. Cells were isolated from hearts of male and female rat pups sacrificed by cervical dislocation at P1 and P2 according to current law of Hessen. The isolated cardiac cells were pooled within one n.

Wild animals

No wild animals were used.

Reporting on sex

As donor mice female as well as male mice are suitable. As recipient mice in our experience, female animals are more tolerable and therefore can be kept in small groups. For rats experiments, mated female Sprague Dawley rats delievered male and female rat pups that were used for isolations.

Field-collected samples

No field-collected samples were used.

Ethics oversight

All animal experiments were executed with the animal welfare guidelines, German national laws, and EU ethical guidelines (Directive 2010/63/EU). For experiments involving rats (cell isolation for cardiac tissue mimetics, Fig. 3a-f, Fig. 8g) protocols were authorized by the competent authority Regierungspräsidium Darmstadt, Hessen, Germany. Experimental mouse models, bone marrow transplantations, left anterior descending artery (LAD) ligation, murine single-cell nuclei (sn) and single-cell (sc) RNA sequencing (Fig. 4, Supplementary Fig. 4a-c, 5a-h, 7d-g) were approved by the Regierungspräsidium Karlsruhe, Baden-Württemberg, Germany. All laboratory animal experimentation described in the manuscript adhere to ARRIVE guidelines 2.0 (ARRIVE Essential 10) for reporting animal research.

Note that full information on the approval of the study protocol must also be provided in the manuscript.
